# Fluorescent Probes for Exploring Plant Cell Wall Deconstruction: A Review

**DOI:** 10.3390/molecules19079380

**Published:** 2014-07-03

**Authors:** Gabriel Paës

**Affiliations:** 1 INRA (French National Institute for Agricultural Research), UMR0614 Fractionation of AgroResources and Environment, 2 esplanade Roland-Garros, 51100 Reims, France; E-Mail: gabriel.paes@reims.inra.fr; Tel.: +33-326-77-36-08; Fax: +33-326-77-35-99; 2 University of Reims Champagne-Ardenne, UMR0614 Fractionation of AgroResources and Environment, 2 esplanade Roland-Garros, 51100 Reims, France

**Keywords:** fluorescent probe, plant cell wall, lignocelluloses, microscopy, enzyme

## Abstract

Plant biomass is a potential resource of chemicals, new materials and biofuels that could reduce our dependency on fossil carbon, thus decreasing the greenhouse effect. However, due to its chemical and structural complexity, plant biomass is recalcitrant to green biological transformation by enzymes, preventing the establishment of integrated bio-refineries. In order to gain more knowledge in the architecture of plant cell wall to facilitate their deconstruction, many fluorescent probes bearing various fluorophores have been devised and used successfully to reveal the changes in structural motifs during plant biomass deconstruction, and the molecular interactions between enzymes and plant cell wall polymers. Fluorescent probes are thus relevant tools to explore plant cell wall deconstruction.

## 1. Introduction

**Figure 1:**
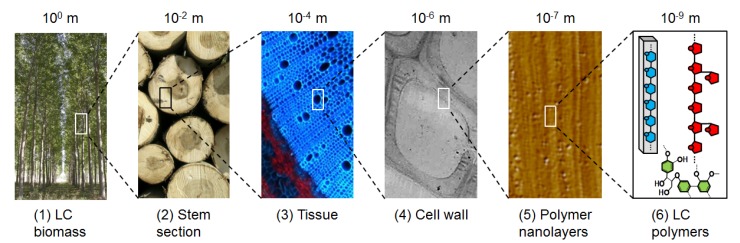
Multi-scale architecture of the PCW. Macroscopic biomass (tree) (**1**) contains different organs; among them stem (**2**) accounts for the largest proportion in weight. Stem is formed by tissues (**3**) composed by cells whose walls (**4**) consist of a matrix of LC nanolayers (**5**) made of cellulose, hemicellulose and lignin (**6**). All pictures are copyright INRA.

The plant cell wall (PCW) is the skeleton of plants: it helps maintaining rigidity against gravity or environmental elements (such as wind, rain), plasticity to permit plant growth, and protection against pathogens. For centuries, wood, which is essentially made up of the cell walls, has been used by mankind for building, producing heat and making tools. More recently, industrial fabrication of paper has reinforced the importance of PCW as a valuable multi-purpose resource. In the last decades, scientists have sought to explore even more deeply the potential of PCW to produce new materials and chemicals. Indeed, PCWs are part of the multi-scale architecture in plants (Figure 1) and are made of three different layers: the middle lamella, rich in pectins and forming the interface between plant cells; the primary cell wall, thin and flexible; and the secondary wall, thick but not present in all cell types. This layer is the most important by weight in the plant wall, and is a highly variable complex network of polymers: 90% of its dry weight is made up of cellulose, hemicellulose and lignin [1], that is why it is more commonly called lignocellulose (LC). Even if the determination of PCW architecture at the molecular scale is the Holy Grail of scientists, global composition is commonly defined: lignocellulosic PCW is composed of different sugars (glucose, xylose, arabinose) and polyphenol molecules. They can thus potentially be used as starting materials for transformation in many synthons and chemicals, by chemical reactions or biological reactions with enzymes or micro-organisms (such as fermentation). Other advantages are that different plant species can be grown virtually at any place in the world, which limits transportation and feedstock dependency: grasses such as wheat, maize are grown in temperate regions, like wood trees; sugar cane in tropical regions, *etc*. Consequently, LC from PCW is considered as a sustainable alternative source of fuels, chemicals and materials that may reduce our current dependency on fossil carbon from oil, natural gas and coal.

Because of its high chemical and structural complexity at different scales [2,3], LC is a recalcitrant substrate whose transformation into valuable products is not currently economically viable. One of the main difficulties to overcome is the low efficiency and high cost of the enzymes required to break down the polymers [4]. Two complementary strategies are underway to find relevant solutions:

- More efficient enzymes: engineering existing enzymes and mining natural diversity of living organisms degrading LC, to create the most efficient biocatalyst cocktail;
- Less recalcitrant LC: pre-treatment with physical, chemical and/or enzymatic steps to make substrates more accessible and increase their digestibility [5]; or altering genetically the PCW architecture [6].

In order to facilitate this process, it has become essential to determine the limitations of enzymatic deconstruction of LC by increasing our knowledge regarding the architecture of LC at multiple scales. To achieve this goal, the dynamics of enzymes and lignocellulose structure should be monitored during enzymatic degradation at different scales, which is still not possible. That is why complementary techniques are needed.

First, many classical biochemical and spectral techniques [7] help determining the primary structure of polymers in PCWs, such as liquid/gas chromatography, FTIR [8], and NMR [9]. Spatial information can be obtained from electron microscopy that provides high resolution images (submicrometric scale) giving structural information, for example on pore size and distribution. Recent advances in tomography have also revealed 3D architecture details, but sample preparation often requires fixed material, not always representative of native hydrated PCWs and provides little or no information on PCW dynamics. Atomic force microscopy for topography has become widely used because it is a high resolution technique, but it only gives access to the sample surface [10]. Regarding dynamical information monitored during substrate catalysis, cellulose particle size changes can be followed *in situ* during enzyme hydrolysis [11], but the resolution is quite low and the technical setup is not straightforward. Overall, essential spatial and dynamical information can only be acquired separately. More importantly, analyses are generally focused on the substrate properties, and very few studies are related to the behaviour of enzymes (shape, activity, interactions) during hydrolysis.

In the course of PCW deconstruction by chemicals and enzymes, molecular processes occur at the nanoscale with nano-, micro- and macro-scale consequences, and thus requires techniques able to follow such events. Imaging and spectral fluorescence analysis are highly sensitive, non-invasive methods that can provide much valuable information on the architecture of PCW and on the interactions and mobility of enzymes in PCW. Endogenous fluorescence of PCW can also be measured (mapping autofluorescence of lignin reveals polymer organisation and interactions [12,13]), but this review will focus on exogenous fluorescence brought about by so-called fluorescent probes. Also, indirect imaging of PCW using antibodies is out of the scope of this review, and interested readers should refer to another article [14].

This review is dedicated to scientists who are not familiar with fluorescence and fluorescence microscopy tools and techniques, and who want to learn how these might be useful for their research applied to the deconstruction of PCW. In the first part, the principle of fluorescence and the different available fluorophores will be presented, in addition to techniques used for imaging. Then, a review of the fluorescent probes used in PCW together with the information obtained will show the advantages of such analysis.

## 2. Fluorophores: Variety and Properties

### 2.1. Definitions of Fluorophores

Fluorophores are molecules having the ability to absorb light energy at an excitation wavelength λ_EX_ to create an excited electronic singlet state. This very short lived state (typically a few ns) undergoes some energy dissipation, so that the light emitted when the fluorophore returns to the ground state has a longer emission wavelength λ_EM_, and is called fluorescent light. The typical fluorescence phenomenon is based on a linear effect: one photon emitted for one photon absorbed. But two-photon absorption can occur when two photons are absorbed simultaneously (which ocurrs in a 10^−18^ s interval) by the same fluorophore: the excitation becomes non-linear. Here it is important to distinguish and define the terms *fluorophore* and *fluorochrome*. Indeed, the latter is the chemical centre of the fluorescence process: for some molecules (in general small ones), the fluorochrome is the entire fluorophore (e.g., fluorescein); while for other, the fluorochrome is only one part of the fluorophore (e.g., fluorescent proteins). For clarity, we will only use the term fluorophore that is more common.

**Figure 2:**
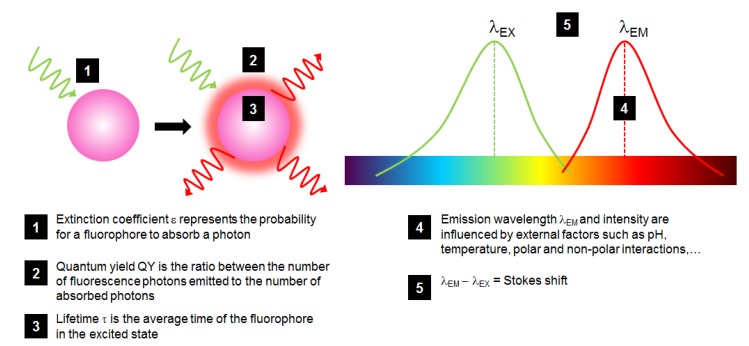
Schematic representation of the fluorescence phenomenon and of the most critical properties of a fluorophore (in pink) excited by a green light and fluorescing red light.

Fluorophores are characterized by different properties that are important to detail in order to make the most adapted choice when using such molecules (Figure 2):

- The probability for a fluorophore to absorb a photon is called the *extinction coefficient* ε, which is at a maximum at λ_EX_;
- The ratio between the number of fluorescence photons emitted to the number of absorbed photons is called *quantum yield* (QY or φ, comprised between 0 and 1), describing how efficient the fluorescence process is;
- The *brightness* of a fluorophore is defined by ε × QY which is a good indicator to compare different fluorophores;
- Fluorescence *lifetime* τ is the average duration in which the fluorophore remains in the excited state: when it returns to the ground state, it follows an exponential decay, in the order of ns. The less long this time is, the better the fluorophore sensitivity is. Lifetime is highly dependent on fluorophore environment and interaction;
- The λ_EX_ – λ_EM_ difference is called the *Stokes shift*: a large one is preferred to distinguish between excitation and emission spectra, a critical measurement of the overlapping of fluorophore excitation and emission spectra for accurate and sensitive analysis.

Fluorescence emission intensity is directly proportional to the excitation intensity. Intuitively, it is reasonable to think that high excitation intensity is useful to detect more easily a fluorophore. Actually, imaging a fluorophore is always accompanied by its photo-destruction called photobleaching. Consequently, excitation intensity must be balanced carefully: high enough to image the fluorophore correctly, but not too much to avoid destruction of the fluorophore during imaging. Photobleaching is a complex photochemical process resulting in the irreversible destruction of the fluorophore. It arises when the fluorophore reacts in its excited state with O_2_ to create highly oxidative reactive oxygen species that bleach the fluorophore and react with other surrounding biomolecules. There are several ways to avoid it: minimizing the sample time exposure and the exciting intensity, preparing samples in mounting systems containing anti-quenches/fading agents, using microscope objectives with high numerical aperture (NA).

Another important phenomenon is fluorescence quenching, that arises when the excited electrons of the fluorophore return to the ground state without emitting light. This happens when the fluorophore directly interacts with another molecule (collisional or dynamical quenching) or with another fluorophore (self-quenching), the latter occurring when the local fluorophore concentration is too high. As a result, fluorescence emission is decreased in intensity (hypochromic effect) and maximum emission wavelength may also slightly increase (bathochromic effect).

Overall, the fluorescence of a fluorophore depends on many physico-chemical parameters (excitation intensity, pH, temperature, electrostatic interactions, *etc*.), so it can only be determined under given conditions. This may seem an inconvenient factor, but it is in fact an advantage: fluorophores can be used as very sensitive sensors, in particular for investigating biochemical processes.

Regarding structural and chemical properties, fluorophores are a very broad class of molecules that comprises organic dyes, fluorescent proteins, quantum dots and other inorganic dyes such as lanthanide complexes. In order to select the most appropriate fluorophore for a given application, several characteristics must be reviewed, in order to make the exogeneous fluorophore compatible with the analysed PCW sample in close relation to the information looked for:

- Spectral properties of the fluorophore: excitation and emission maximum wavelengths and bandwidths, brightness, photostability;
- Chemical properties: solubility, stability, ability to be conjugated to another biomolecule;
- Physical properties: molecular weight, size, oligomerisation properties.

The goal of the sections below is not to present an exhaustive panorama of existing fluorophores, but rather to focus on those used in the PCW deconstruction area. Interested readers should for example refer to the review article on fluorophores by Hötzer *et al.* [15]. The most important properties of fluorophores are gathered in Table 1.

### 2.2. Small Organic Fluorophores

The dye fluorescein is probably one the most common fluorophores in use today. Its very high molar absorptivity at the wavelength of the argon laser (488 nm), large fluorescence QY, and water compatibility make it a very useful and sensitive fluorescent label. Fluorescein is commercially available as many derivatives, such as fluorescein isothiocyanate and fluorescein succinimidyl ester, that can be covalently attached to macromolecules and to amino acids [16]. Limitations of fluorescein are a quite low photostability (which actually can be advantageous, see FRAP section below) and fluorescence pH sensitivity, fluorescence being largely reduced below fluorescein pKa (ca. 5.0) [16].

**Table 1:** Properties of the principal fluorophores used for PCW investigation.

	Fluorescein	Tetramethylrhodamine	Alexa Fluor^®^ 488	GFP	QD (CdSe/ZnS)
Maximum Excitation/Emission Wavelengths (nm)	490/520	544/573	495/519	395/504	405/605
QY	0.90	0.28	0.92	0.79	0.40
ε (M^−1^·cm^−1^)	80,000	99,000	73,000	30,000	2,400,000
Brightness	72,000	27,700	67,000	21,600	1,000,000
Fluorescence Lifetime (ns)	4.1	1.6	4.1	2.7 and 3.3	>10
Hydrodynamic radius (nm)	0.4	0.4	0.4	1.7	10
Molecular Weight (Da)	389	444	643	~28,000	N/A

Rhodamine dyes such as tetramethylrhodamine (TMR) have excitation maxima beyond 520 nm and emission going up to 700 nm, so they can be easily excited by the 543 nm green He-Ne laser or by the 546 nm spectral line from mercury-arc lamps. TMR’s spectral properties are not affected by changes in pH between 4 and 10 which is of great interest for biological applications. Compared to fluorescein, its QY is lower but it is more photostable. TMR is more hydrophobic, resulting in a tendency to aggregate in aqueous solution. Consequently, fluorescence emission can be reduced because of self-quenching, and the complex emission spectrum obtained makes it difficult to determine labelling (see the section below). Like fluorescein, TMR is available as isothiocyanate or succinimidyl ester derivatives for easier bioconjugation.

Alexa Fluor^®^ dyes are a class of widely used commercial fluorophores derived from fluorescein and spanning the visible and infrared spectrum. They are generally more photostable and brighter than they parental fluorophore, but are also much more expensive. One great advantage is that Alexa Fluor^®^ fluorophores are available in a variety of activated derivatives for easy labelling. BODIPY^®^ can also be an alternative, with spectral properties and variety close to Alexa Fluor^®^ but lacking ionic charge, so that fluorescence properties are much less dependent on pH and polarity (readers should consult the Molecular Probes catalogue for an exhaustive presentation of the available dyes).

### 2.3. Fluorescent Proteins

Fluorescent proteins (FPs) are nearly all derived from the jelly fish *Aequorea victoria* green fluorescent protein (GFP) [17] or from *Discosoma* sp.. GFP has a barrel-like structure hiding a 4-(*p*-hydroxybenzylidene)-5-imidazolinone fluorochrome. Genes coding for FPs are available commercially in a large array of spectral classes, excitation wavelength ranging from UV to far-red [18], after evolution of GFP has been performed through molecular biology mutations. Possibilities to produce genetically encoded FPs, in addition to their diverse spectral properties, make FPs relevant biologically-compatible fluorescence reporters. Selection of most relevant FP depends on parameters such as QY, brightness, photostability, which vary a lot from a FP to another. A complete guide for choosing FPs has been proposed [19]. But FPs can have some drawbacks: first, they are much larger than small organic fluorophores (more than 20 kDa compared to less than 1 kDa), possibly preventing some interactions by size exclusion or target inaccessibility. Second, some FPs have a tendency to make oligomers: in addition to the size increasing, this can disturb the function of the protein to which it is attached. Recent successful researches succeeded in creating monomeric FPs [20].

### 2.4. Inorganic Fluorophores

Quantum dots (QDs) are semiconductor nanocrystals whose spectral properties are size-dependent: the smaller they are, the smaller the excitation and emission wavelengths are. Lifetime is also proportional to their size. They are made of an inner core, a shell and surface coating. Common composition of QDs is CdSe for the core and ZnS for the shell, which makes this assembly highly hydrophobic and not biocompatible. A coating is applied to incorporate ionizable chemical functions or polyethyleneglycol chains, increasing stability and water solubility, and to facilitate functionalization with other biomolecules. QDs have unique optical and chemical features: high brightness, long Stokes shift (no or low overlapping between excitation and emission spectra), chemical and photostability even in relatively harsh environments, but they are large (hydrodynamic diameter of 5–20 nm) compared to organic fluorophores. They have also a good potential for FRET measurements; because of their broad excitation spectrum, they can only be considered as donor and not as acceptor.

### 2.5. Fluorophore Imaging

#### 2.5.1. Sample Preparation

PCW samples can be native or pre-treated biomass. In the former case, whatever the plant species is (wood or field plants such as wheat, maize, sugar cane, miscanthus, *etc*.), some fibres can be obtained or thick sections (between 10 and 60 µm) are routinely prepared by cutting slices with a microtome. In the case the biomass has been ball-milled or steam-exploded and turned into powder, some sections sometimes of course cannot be prepared.

The conditions used when applying the fluorescent probe onto the PCW sample are critical but cannot be generalised. Of course, the first goal is to keep the sample intact and stable during imaging. Usually, fixed samples are generally mounted in a glycerol-based buffer between a glass slide and a cover slip. The buffer and the pH must be tested to be compatible both with the sample and the fluorescent probe, so that the lignocellulosic architecture is not modified, and the fluorophore properties are not altered. Consequently it remains as bright as possible (see Section 2). Caution must be taken regarding mounting medium that may contain anti-fading and/or anti-photobleaching molecules. These are very convenient when simply imaging PCW samples, but can be detrimental when photobleaching techniques are used. The optical path between sample and microscope objective (medium/cover slip/oil or air) must also be constant, otherwise observations become impossible.

Observing fluorescent probes can be particularly challenging in materials already containing endogenous fluorescent molecules. In PCWs, autofluorescence mainly originates from the aromatic polyphenolic structure of lignin. Indeed, when excited at common wavelengths such as 343 nm or 488 nm, broad emission spectra are generally observed in green or blue [13]. Different well described methods for discriminating autofluorescence have been set up by optimizing instrumentation and correcting images after measurement (for a comprehensive review see [21]).

#### 2.5.2. Microscopes

Microscopy imaging allows a spatial, spectral and temporal investigation of the sample. Once prepared, mounted sample comes in contact to the microscope objective lens. Lens is characterized by its magnification (usually from ×10 to ×100) and its numerical aperture (NA). The latter is directly correlated to the number of photons that can be collected from the sample, so a high NA is preferred.

Depending on the thickness of the sample, different types of microscopes may be used:

- A wide-field microscope for thin samples, but axial resolution is limited;
- A confocal laser scanning microscope (CLSM) for thicker samples (100 µm and below) since this instrument can image sections of samples in the axial direction with a resolution depending on the excitation wavelength λ_EX_ and the objective NA (theoretical axial resolution is 1.4λ/NA^2^);
- A multiphoton microscope. In order to increase spatial resolution, a two-photon absorption technique can be set up. Indeed, a fluorophrore can be equivalently excited by a photon with a wavelength λ_EX_ or by two photons with a wavelength of approximately 2λ_EX_. As a result, the excitation volume of the fluorophore is limited to the focal point and is not spread. Since the excitation wavelength is doubled, it is twice less energetic: this technique limits sample autofluorescence and is much less damaging. Moreover, longer wavelengths are less absorbed by the sample and are also less affected by light scattering, allowing a better penetration in the sample. It is also particularly convenient when fluorophore observation requires UV excitation because two-photon infra-red light is enough to image sample, limiting sample and fluorophore damaging (for a review on multiphoton microscopy see [22]). However, multiphoton microscopy necessitates a femtosecond pulsed laser, which is a very expensive instrument.

A very useful checklist for optimizing images for quantitation has been proposed [23], for increasing signal and decreasing noise and background.

#### 2.5.3. Microscopy Imaging Techniques

In PCW investigation with fluorescent probes, different imaging techniques can be used depending on the goal of the analysis:

- For mapping fluorescent probes in samples, simple imaging techniques are routinely applied;
- For determining the diffusion and interaction processes of fluorescent probes, photobleaching techniques are very useful. They are performed in a CLSM, using a fast scan imaging (compromise between spatial and temporal information) to minimize laser intensity during imaging (except for the bleaching step);
- For evaluating some interaction processes at molecular scale, measuring the energy transfer between the fluorescent probe and another endogenous fluorophore from the PCW is valuable but difficult to set up correctly.

**Figure 3:**
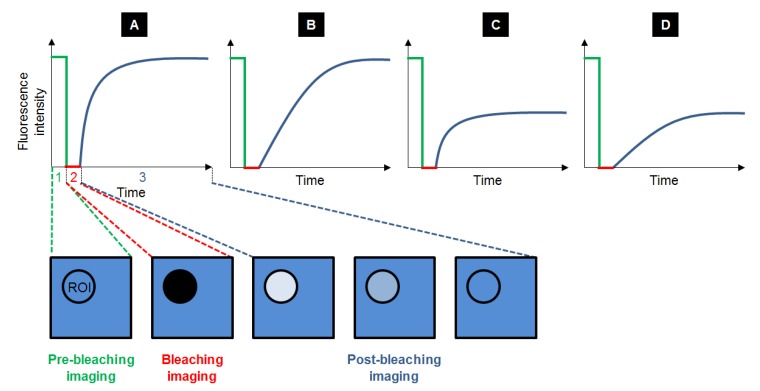
Typical recovery curves obtained after FRAP experiments: the fluorescence intensity is measured before (step 1, in green), during (step 2, in red) and after photobleaching (step 3, in blue), while equilibrium is taking place. Several cases can be observed depending on the velocity and the amplitude of the recovery. Recovery can be fast with a maximal recovery (**A**), or slow with a maximal recovery (**B**). It can also be fast with a limited recovery (**C**) and even slow with a limited recovery (**D**).

Fluorescence Recovery After Photobleaching (FRAP) is the most ancient fluorescence technique to study dynamic processes, by unravelling compartmentalization of the specimen studied, diffusion of the fluorescent probes and their interaction with the system. Typically, a FRAP experiment is divided in three steps. First, a circular region of interest (ROI) in the sample is selected and its pre-bleaching fluorescence intensity is recorded. Then, the ROI is bleached with a high power laser beam: the fluorophores inside the ROI are irreversibly destroyed. Subsequently, the surrounding fluorescent molecules that can freely diffuse into the ROI increase the ROI fluorescence, so that the fluorescence recovery is recorded until it reaches a plateau (Figure 3). The fraction of probes that can exchange between the non-bleached area and the bleached ROI is called the mobile fraction (MF), whereas the fraction that cannot exchange is called the immobile fraction (IF). FRAP gives access to the diffusional exchange occurring between the bleached ROI and the surrounding area. Practically, the fluorescence of the ROI is recorded during the full FRAP experiment to give a kinetic plot of fluorescence *vs.* time. These raw data often need to be corrected to take into account the fact that the whole sample can be partially bleached during imaging, so intensity in a ROI far from the bleached ROI is also recorded to obtain the real intensity recovery. The data points obtained are generally fitted with a more or less sophisticated exponential equation, whose parameters will give access to:

- The mobile and immobile fractions (MF and IF);
- The half-life time τ_1/2_, which is the time needed to reach half of the maximum/final recovery intensity. Using Equation (1) [24], where *r* is the radius of the bleached ROI, gives access to the diffusion constant *D*:

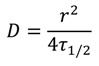
(1)

Depending on the recovery curve shape (Figure 3), some qualitative information can be obtained regarding the probe mobility and the structure of the system. In order to go into more investigation, fitting FRAP recovery curves to diffusion and/or binding models can reveal kinetic details on the behaviour of the fluorescent species and their interaction to the medium. Indeed, probe mobility is determined both by diffusion and binding interactions, both phenomenon influencing each other. Three different scenarios can happen [25]: the diffusion is dominant, no binding occurs; the diffusion is much slower than the binding process (effective diffusion); the diffusion is slower than binding (binding is dominant). Distinction between the two latter processes can be easily done by using different ROI sizes for bleaching to determine whether the FRAP recovery changes [26]. In the case where binding association is much faster than the diffusion time, diffusion appears slowed down (effective diffusion *D*_eff_) compared to diffusion in the absence of binding (*D*). The ratio between *D*_eff_ and *D* yields the pseudo-equilibrium constant k*_on_ / *k*_off_, which is representative of the affinity of the fluorescent probe:

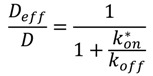
(2)


Detailed protocols for beginning FRAP experiments and data processing are available for qualitative and quantitative analyses [27]. An easy way to validate one FRAP protocol is to measure the diffusion of a rather large fluorescent molecule (e.g., a commercial fluorescent dextran) in water or appropriate buffer and to compare it to data available in literature. The important point to consider in FRAP experiments is that obtaining absolute diffusion values is unrealistic; but employing the same protocol for a series of probes in the same medium can give a quite accurate relative comparison of the behaviour of the probes.

Another useful method with probe fluorescence is Förster Resonance Energy Transfer (FRET). It is a process by which excited-state energy is transferred directly from one fluorophore (the “donor”) to another nearby fluorophore (the “acceptor”) through near-field electromagnetic dipole interactions. The three basic requirements for measuring an efficient energy transfer from the donor to the acceptor are: i/ they must be in close proximity, ii/ they must have a favourable dipole-dipole alignment, and iii/ they must share significant spectral overlap. Then, the quantification of FRET signals in microscopic images can give nano-scale measurements of the spatial relationship between the fluorophores labelling molecules [28].

FRET efficiency *E* is dependent on the distance *R* between the acceptor and donor and on *R*_0_, which is the distance for which *E* = 50% (Equation (3)):

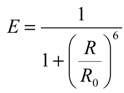
(3)


*R*_0_ is usually in the range of 2.0 to 7.0 nm [29] and depends on several factors: donor QY, acceptor extinction coefficient, spectral overlap between donor emission and acceptor excitation, wavelength, and an orientation factor. In addition, donors with long lifetime maximises FRET probability to occur, while acceptors with short fluorescence lifetime are useful since they limit FRET saturation. It is generally admitted that a FRET can only be measured if its efficiency is at least 10%, so that FRET does not happen when *R* > 10 nm.

The FRET efficiency is generally estimated by measuring fluorescence intensity changes of both donor and acceptor (spectral imaging FRET), or fluorescence lifetime decrease of the donor (FLIM-FRET). Spectral imaging FRET can be detected quite simply in a spectrofluorometer or in a CLSM by measuring changes in spectral intensity: if a FRET occurs, the intensity of the donor emission decreases while that of the acceptor increases. This method is quite simple, rapid, but requires analysing three samples (the donor, the acceptor and both during FRET), and is only qualitative since spectral bleed-through happens due to the overlapping of emission spectra.

FLIM-FRET method is based on the measurement of the fluorescence lifetime of the donor alone (τ_D_) and of the donor in the presence of the acceptor (τ_DA_). Only a fraction of the donors undergoes a FRET with acceptors, resulting in a bi-exponential decay curve for the donor instead of a mono-exponential. FRET efficiency is directly correlated to the ratio between the lifetimes:

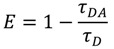
(4)


In comparison to spectral imaging FRET, FLIM-FRET gives access to a quantitative measurement of both the FRET efficiency and of the distance between donor and acceptor, independently of the fluorophore concentration. As fluorescence lifetime is generally only a few nanoseconds, it can only be measured by using a femto-second pulsated laser equipped on a CLSM to perform a time-correlated single photon counting (TCSPC), which is a quite complex experiment and analysis to set-up.

Overall, FRET can bring nanoscale information regarding interaction and dynamics of molecules. But it is important to notice that several conditions must be met to allow an electronic transfer between the fluorophores. Consequently, two molecules bearing compatible fluorophores for FRET may interact without triggering a FRET, but if no FRET occurs, it can also mean that there is no interaction between the probe and the polymer. So FRET interpretation must be made with caution.

For a comprehensive presentation of FRAP and FRET analysis techniques, please refer to the following reviews [30,31] and to for remarkable illustrative schemes [32]. Parameters of digital image acquisition that affect the accuracy and precision of quantitative fluorescence microscopy measurements are smartly described in the following article [23].

## 3. Fluorescent Probe Families

### 3.1. Definition

**Figure 4:**
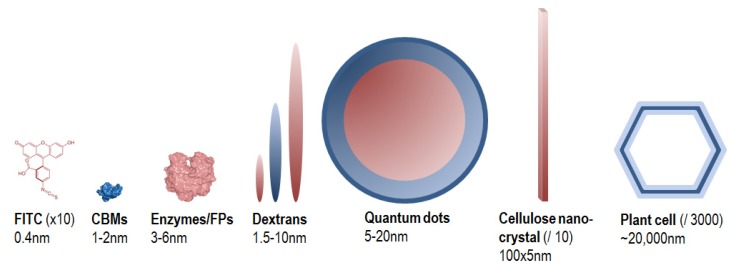
Size comparison of fluorophores, probes and PCW elements. Because of large size differences, the PCW elements are not at the same scale.

Fluorescent probes are fluorophores taken alone or conjugated to a biomolecule, and whose fluorescence signal is exploited for the visualization and the analysis of dynamical phenomenon occurring *in situ*. They are designed because of their specific properties such as size, substrate/ligand specificity and affinity, catalytic activity, *etc*., in order to bring relevant chemical, physical and/or spectral information regarding the sample analysed. Figure 4 gives an overview of size comparison between fluorophores, probes and the objects that they are likely to target in the PCWs.

When a fluorophore needs to be conjugated to a biomolecule, the labelling step is a critical point to control: the presence of the fluorophore must not alter the biomolecule properties, or to a very little extent, and labelling must be adapted to the fluorophore/biomolecule couple and to the desired application. Many parameters have to be checked for characterizing the changes occurring in the conjugate: size, hydrophobicity, chemical structure as well as charge. When the biomolecule is an enzyme, an activity assay is particularly useful for testing the conjugate. But when the biomolecule has no catalytic activity, other tests must be performed for example to assay some conjugate changes in size (by light scattering analysis) or in affinity (by fluorescence spectroscopy or isothermal titration calorimetry). Overall, there are three main approaches to prepare fluorescent-biomolecule conjugates (for a comprehensive review of bioconjugation techniques see [33]):

- Covalent labelling: a covalent bond is created between the fluorophore and the biomolecule. Most common reactions are between the isothiocyanate function in activated fluorophores such as fluorescein and amine functions of amino acid residues in proteins which can lead to the creation of a covalent bond at pH 9.0 or above. Functionalized QDs with carboxylate or amine functions can conjugate to primary amines or thiol functions;
- Coordination labelling: a coordination between poly-histidine motifs in proteins can strongly conjugate to metals (Co, Cu, Fe, Mn, Ni, Zn) such as those found in QDs;
- Heterologous expression: in the case of a recombinant biomolecule like protein, the gene sequence coding for a fluorescent protein can be added before or after that of the biomolecule, so that the two molecules are translated on the same polypeptide.

The labelling choice is based on the properties of the fluorophore selected (with a specific, strong, stable fluorescence signal) and of the biomolecule. In the case of the covalent labelling procedure, it is strongly influenced by several parameters: pH, buffer type and concentration, biomolecule type, biomolecule-fluorophore ratio, reaction time, *etc*. So only a general protocol can be proposed by fluorophore suppliers and must be adapted to the fluorophore/biomolecule that will be conjugated. The biomolecule/fluorophore ratio in the reaction mixture may be the most critical parameter because it determines the degree of labelling (DoL) which is the average number of fluorophore attached to a biomolecule after conjugation, calculated by absorbance measurements. In general, a high DoL is desirable to increase fluorescence imaging sensitivity. But over-labelling can cause fluorescence quenching (see Section 2.1) resulting in fluorescence emission loss or solubility decreasing. Moreover, there is no explicit correlation between fluorescence intensity and DoL.

The term “average” related to the DoL is important because after labelling, the reaction will lead to a heterogeneous mixture of biomolecule-fluorophore conjugates and it is nearly impossible to have a perfectly homogeneous population for two main reasons:

- During the conjugation reaction, the activated fluorophore may react with different chemical sites on the biomolecule, unless there is only one available, which is quite rare;
- Even if a gel-filtration or dialysis of the conjugating reaction mixture is performed to purify the reaction product, the size difference between the biomolecule and the biomolecule/fluorophore conjugate can be very small (it is particularly true when the biomolecule is much larger than the fluorophore).

Nevertheless, a general procedure can be applied for protein labelling, by determining the extinction coefficient of the biomolecule and of the fluorophore, the absorbance at 280 nm of the conjugate and at the maximal absorbance for the fluorophore. Since the fluorophore usually absorbs light at 280 nm, a correction factor is used and is generally provided by fluorophore providers (for instance Life Science, Sigma, Thermo). Table 2 summarizes the advantages and drawbacks of main fluorophore classes used in PCW studies, with several examples illustrating their application being given in the following section.

### 3.2. Small Fluorescent Probes and QDs

Historically, several small fluorescent molecules binding to polysaccharide have been used to localize polymers in PCWs, for example:

- Calcofluor white binds cellulose but not with a high specificity compared to other plant polymers [34];
- Congo red binds more strongly xyloglucan compared to cellulose but also binds proteins [34];
- Phloroglucinol stains lignin in red proportionally to the lignin content;
- Safranine binds lignified tissues in plants and wood, so its red fluorescence is a relevant sensor for differentiating cellulose-rich and lignin-rich cell walls [35];

Newly developed fluorophores exhibit spectral characteristics changes such as fluorescence emission increasing or shift when bound to some polysaccharides. For example, a comprehensive analysis of binding of two fluorophores, Solophenyl Flavine 7GFE and Pontamine Fast Scarlet 4B (S4B), has revealed they bind selectively to cellulose and xyloglucan [34]. The aromatic structure of these fluorophores resembles that of polysaccharide binding sites encountered in proteins and enzymes and might explain their interaction with polysaccharides. Nonetheless, exact molecular interactions are still to be discovered. S4B was used to label *Arabidospis* cell walls, revealing the cellulose distribution and orientation during cell wall expansion. The same strategy has been transferred for the observation of cellulose microfibrils in pine tracheids [36], but overall, molecular sites involved in the chemical interactions between fluorophore and PCW polymers have not been described thoroughly. So more investigation are required, in particular extensive binding assays of fluorophores with PCW samples like those previously performed with antibodies.

**Table 2:** Comparison of the major properties of fluorophore classes used for investigating PCWs. Properties are highlighted in 3 different colours to indicate, in the case of PCW imaging, if they are advantageous (in green), detrimental (in red) or in-between (in orange). Please note that these are general trends and should be adjusted to specific applications.

		Small Fluorescent Molecules	Fluorescent Proteins	Quantum Dots
Spectral properties	Brightness	Low to medium	Medium	High
	Photostability	Low, prone to photobleaching	Low, prone to photobleaching	High
	Stokes shift	Narrow	Narrow	Large
	Lifetime	Long	Long	Short
	Available at various excitation/emission λ	Yes	Yes	Yes
Physical properties	Size (hydrodynamic diameter)	Small <1 nm	Medium >3 nm Prone to oligomerization	Large >10 nm
Chemical properties	Preparation	Commercial or lab dyes	Commercial genes	Commercial or lab
	Labelling	Several derivatives can react with many functions DoL can be difficult to determine	Recombinant cloning DoL is known by construction	Difficult to control with accuracy
	Conjugate stability	High	Medium	Weak
Other		Hydrophilic or hydrophobic Generic small fluorophores are cheap		Blinking (decreases QY), possibly toxic

**Figure 5:**
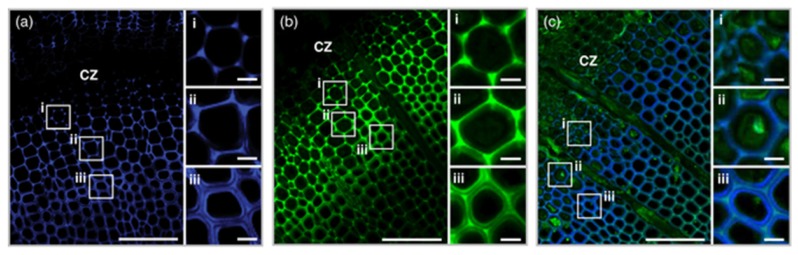
Transverse sections of *Pinus radiata* stem labelled with two fluorescent probes (DMAC-tagged monolignol probe, DG; NBD-tagged monolignol probe, NG) showing developing xylem tissues around the cambial zone (CZ). (**a**) Fluorescence of DG (purple); (**b**) fluorescence of NG (green); (**c**) fluorescence of DG (purple) and autofluorescence (green). Scale bar = 100 µm in wide-field and 10 µm in magnified images. Figure from [38].

Incorporation of labelled biomolecules that can be metabolized by plant cells *in vitro* or *in planta* is another relevant strategy, exploited for imaging some dynamical processes. For example, xyloglucan labelled with sulforhodamine are incorporated in cell walls and allows following trafficking of the oligosaccharide [37]. In recent advances, monolignols conjugated to fluorophores have been metabolized by *Arabidopsis* and *Pinus* samples in order to follow lignification process *in planta*. This allows direct visualization and localization of PCW lignification, fluorescent probes are readily distinguishable from the other cell wall components such as polysaccharides and pre-existing lignins [38] (Figure 5).

QDs can be conjugated to biomolecules but are also used directly as fluorescent probes. Interactions of CdSe QDs with conifer samples were studied: QDs interact with cellulose via hydroxyl groups and with lignin via conjugated molecules, indicating that QDs are probes with low ligand specificity [39]. Only advantage of QDs taken alone is their high electron density, so they can be observed directly in electron microscopy, which allows photonic and electronic microscopy correlation microscopy analysis. When conjugated to other biomolecules, their large size can alter the properties of biomolecules to which they are conjugated, but it also permits to graft several biomolecules to a single QD.

### 3.3. Polymers as Fluorescent Probes

Dextrans labelled with FITC are among the most common fluorescent probes. Contrarily to the small fluorescent probes, they are not used for mapping. Rather, they are very advantageous since they are cheap, chemically and enzymatically stable, pretty inert (low interaction with proteins or polysaccharides), and available in various molecular weights ranging from 5 to 2,000 kDa which is useful to explore the impact of size on dynamic processes. Diffusion of a set of dextran-FITC was performed to investigate the porous structure of cellulose filter paper [40]. This study provided a quantitative measure of the fraction of the substrate structure that is accessible to the probes based on their relative size. A similar work has been done with dextran-FITC in bio-inspired PCW assemblies with variable water content containing cellulose nano-crystals and arabinoxylans in different proportions, in order to hierarchize the different parameters influencing diffusion measured by FRAP [41,42]. Results showed that the covalent and non-covalent interactions between polymers have a strong impact on the assembly architecture and thus on probe mobility. In addition, the fluorescent probe size and the water content are the most critical parameters affecting the probe progression: little variations of these parameters may have a large effect. Even if performed on simplified LC systems with non-biomass-related probes (dextran), both studies show how modelling studies can bring valuable information on the micro-scale diffusion of PCW degrading enzymes.

In order to investigate the non-productive adsorption of cellulose degrading enzymes on lignin, which is one reason for reduced conversion of LC, some FRET measurement have been performed between polyethyleneglycol (PEG) labelled with rhodamine and lignin from pine cell wall [43]. Fluorescence quenching and FRET demonstrated the binding of PEG onto lignin. It suggests that more exposed and accessible lignin from porous secondary cell wall could be responsible for non-productive binding of enzymes compared to less accessible lignin from the middle lamella.

### 3.4. Proteins as Fluorescent Probes

#### 3.4.1. Proteins with no Catalytic Activity: CBMs

Carbohydrate binding modules (CBMs) are defined as “a contiguous amino acid sequence within a carbohydrate-active enzyme with a discrete fold having carbohydrate-binding activity” and classified in more than 60 families in the CAZy database [44], depending on their binding specificity against polysaccharides. CBMs are appended to enzyme degrading PCW, the generally recognized roles of CBMs are (i) to increase effective enzyme concentration on the substrate surface by targeting some structural motifs, resulting in an enhancement of hydrolysis of insoluble substrates [45]; (ii) to disrupt crystalline substrate by weakening and splitting hydrogen bonds, which is part of the more global lignocellulosic substrates amorphogenesis [46]. Thus, CBMs play a critical role in the recognition of PCW components [47], that is why they have been advantageously utilized as molecular probes for the analysis and detection of polysaccharides in PCWs [48], in order to reflect the micro-heterogeneity of polysaccharides. Since CBMs are proteins, they can routinely be produced and purified, then adding a fluorescent protein, or conjugated to a fluorophore.

**Figure 6:**
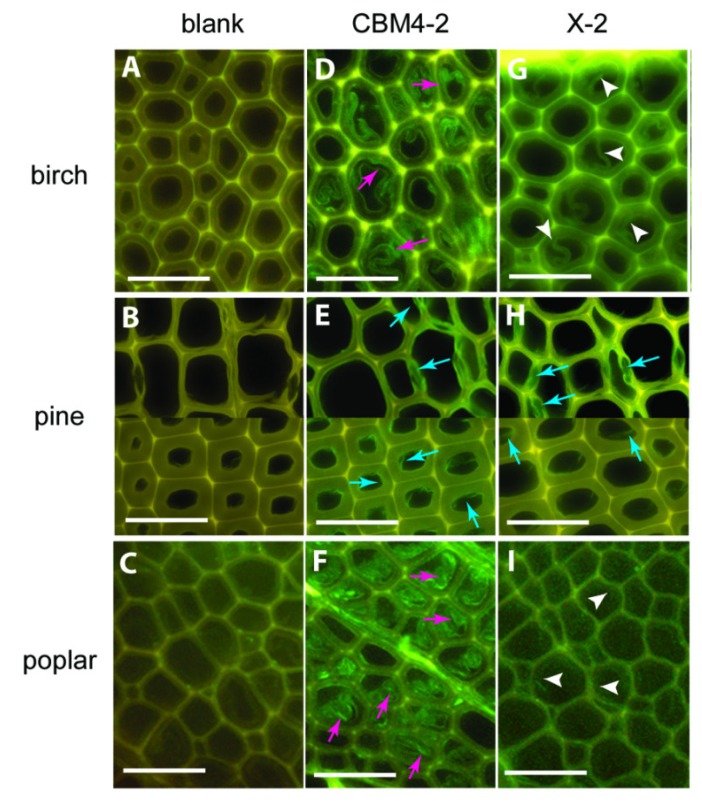
Differences in binding of CBMs to various wood sections: CBM4-2 (D-F) and X-2 (G-I) in birch, pine and poplar. Negative control staining of wood sections (blank, **A**–**C**) with mouse anti-His antibody and FITC-conjugated anti-mouse IgG. Scale bar = 50 µm. Figure from [50].

As fluorescent probes, CBMs usually serve for localising some PCW components and their accessibility in their native state or after some biomass pre-treatments. A family 1 CBM labelled with FITC was used as a probe to detect cellulose in various lignocellulosic materials [49], while synthetic xylan-binding CBMs attached to FITC interacted differently to various wood type sections [50] (Figure 6). Some family 3, 6 and 20 CBMs were also grafted with QDs, using the strong coordinate covalent interaction between their His-tag and the ZnS shell of the QD, in order to compare the cellulose localization of fibre and primary cell walls [51]. Fluorescent proteins have also been conjugated to CBMs. Some family 3 CBMs engineered with fluorescent protein DsRed (RFP) and imaged in corn fibre have shown the impact of pre-treatment on the cellulose accessibility, in particular when hemicellulose or lignin fractions are removed [52]. Family 3 and 28 CBMs expressed with cyan fluorescent protein were used to determine crystalline and non-crystalline cellulose exposure in differently pre-treated wood, to estimate hydrolyzability and the reaction sites where enzymes may bind and begin hydrolysis [53]. Finally, a family 1 CBM from *T. reesei* cellulase CBHI was coupled to GFP to study binding on *Valonia* cellulose crystalline microfibrils. Examination of the fluorescent probe by defocused orientation and position imaging revealed that the CBM binds specifically to hydrophobic crystalline planar faces [54]. The same authors tracked a family 2 CBM conjugated to a QD, in order to be able to follow a bright fluorescent single particle. Contrarily to the QD tested alone whose movement was random, the fluorescent CBM exhibited a linear motion along the cellulose fibre [55].

CBMs have also demonstrated their ability to uncover some enzyme dynamics. For example, the surface diffusion of a family 2 CBM naturally associated to a cellulase and conjugated to FITC was followed by FRAP on microcrystalline cellulose sheets. It showed that when alone, CBM is adsorbed irreversibly; but when it is linked to the enzyme, the processivity of the conjugate is detected: this demonstrates that surface diffusion of CBMs does not limit substrate catalysis [56].

#### 3.4.2. Proteins with Catalytic Activity: Enzymes

Like CBMs, fluorescent enzymes can help mapping substrate, after their catalytic activity has been turned off, for example by mutating one catalytic residue. In this frame, a genetically inactivated GH11 endoxylanase was labelled with Alexa Fluor^®^ 488 to probe arabinoxylan in various layers of cereal cell walls. Due to the high specificity of the enzyme for low substituted arabinoxylan, imaging was performed with a great precision, depending on arabinose substitution [57].

Fluorescent enzymes are also very useful to follow *in situ* catalysis and understand degradation mechanisms. The *Trichoderma reesei* Cel7A was labelled with the Alexa Fluor^®^ 647 dye to follow binding on bacterial microcrystalline cellulose during depolymerisation. The resulting kinetic model revealed that exposing new binding sites is an important rate-limiting step in the hydrolysis of crystalline cellulose [58], which has a strong impact for rationalising biomass pre-treatment.

Following the diffusion of fluorescent enzymes is also highly relevant. The surface diffusion of *Thermobifida fusca* cellulases Cel5A, Cel6B and Cel9A conjugated to Alexa Fluor^®^ 488 or Alexa Fluor^®^ 647 was followed on bacterial microcrystalline cellulose by FRAP and single molecule tracking. Cellulases exhibited complex, non-steady surface motions, with different diffusion states: immobile state, steady sliding motion (2D diffusion on the cellulose surface) and short-lived “hops” (quick move over hundreds of nanometers, which corresponds to unbinding, solvent diffusion followed by binding) [59]. In particular, loss of catalytic activity during cellulose depolymerization was caused by cellulases adsorbing irreversibly onto cellulose [60]. Likewise, FRAP analysis was used to evaluate the importance of catalytic activity and binding of a GH11 xylanase labelled to Alexa Fluor^®^ 488. By comparing the mobility of the native enzyme and the genetically inactivated enzymes on two isolated xylan substrates, authors showed that xylanase mobility was dependent on both substrate hydrolysis and targeting: mobility was inversely correlated to affinity [61].

Only a few FRET measurements have been carried out with fluorescent enzymes. A proof of concept was proposed by measuring interactions between carboxymethyl-cellulose (CMC) labelled with fluorescein (donor) and a cellulase conjugated to Alexa Fluor^®^ 594 (acceptor) by spectral analysis [62]. Even with experimental pitfalls due to non-uniform labelling and the spectral FRET measurement which is not quantitative, authors have demonstrated that lower temperature favours the cellulose binding onto CMC. They also used FRET to investigate co-localization of cellulases on cellulose fibre, confirming that these cellulases tend to bind to their substrate by following each other [63].

## 4. Conclusions and Perspectives

Joint progresses in fluorescence imaging accessibility (fluorescence and confocal microscopes) and in fluorescent probes availability (tens of fluorophores and labelling protocols) have permitted the emergence of routine use of fluorescent probes for investigating PCW architecture. In comparison to other techniques, fluorescence imaging is very fast and sensitive, and techniques ranging from steady-state to time-resolved imaging and energy transfer offer a wide panel of tools, as exposed in this review. Nonetheless, light microscopy is by definition limited by its optical resolution (in CLSM, lateral resolution is 180–250 nm, while axial resolution is 500–700 nm). In recent years, new super-resolution techniques either based on tailored illumination, non-linear fluorophore responses, or the precise localization of single molecules have emerged (for a detailed review see for example [64]). Main advantages are to reach lateral resolution around 20 nm, thus uncovering new details of biological phenomenon as already demonstrated in biomedical sciences. But these techniques often require the use of specific fluorophores, no more than two different fluorophores simultaneously, and consist in state-of-the-art microscopy sometimes difficult to set-up. This should be a path to follow to improve our knowledge in PCW deconstruction, like recently demonstrated with the first super-resolution images of cellulose bundles in the PCW [65].

Another interesting recent development in fluorescence regards the photoactivable fluorescent proteins. They are capable of pronounced changes in their spectral properties in response to irradiation with light of a specific wavelength and intensity. Some convert from a low (dark) to a bright fluorescent state (photoactivation), whereas some others change fluorescence colour (photoswitching or photoconversion) [66]. Since fluorescent protein observation does not necessitate continuous excitation, they are less prone to photo-inactivation. When fused to proteins of interest (such as enzymes or CBMs), they represent relevant tools for accurate protein tracking and mobility measurements, FRET measurements and some super-resolution techniques which require photoactivable proteins [67]. All these exciting techniques will for sure be applied to PCW deconstruction studies leading to a bright future for fluorescent probes.
